# Comparison Between Oral Anticoagulation and Left Atrial Appendage Occlusion in the Prevention of Stroke With Regard to Non-Valvular Atrial Fibrillation

**DOI:** 10.7759/cureus.10437

**Published:** 2020-09-14

**Authors:** Osama Sandhu, Zarmeena Aftab, Adarsh Thomas Anthony, Shermeen Rahmat, Safeera Khan

**Affiliations:** 1 Internal Medicine, California Institute of Behavioral Neurosciences & Psychology, Fairfield, USA; 2 Family Medicine, California Institute of Behavioral Neurosciences & Psychology, Fairfield, USA; 3 Family Medicine , California Institute of Behavioral Neurosciences & Psychology, Fairfield, USA

**Keywords:** left atrial appendage occlusion, oral anticoagulation, stroke, non valvular atrial fibrillation

## Abstract

In the past, the most common type of atrial fibrillation leading to stroke was valvular; this was predominantly due to the prevalence of rheumatic fever, but with the advent of better-hospitalized care, the cases of valvular atrial fibrillation declined. In recent years, there has been an increase in cases of stroke due to non-valvular atrial fibrillation. Stasis of blood in the left atrial pouch leads to coagulation and thrombi formation, which may lead to stroke. Oral medication or mechanical intervention can prevent thrombi formation. Both oral anticoagulation and left atrial appendage occlusion (LAAO) have been compared to see which has better outcomes. It was observed that LAAO has greater efficacy, but with time throughout a couple of years, no considerable difference was seen when compared to warfarin. Most of the long-term randomized controlled trials have been performed with the Watchman^®^ device. Although the Lariat and Amplatzer LAAO devices have also shown favorable outcomes, there is still a deficiency when it comes to trials of high-quality evidence using these devices as an intervention. Dual therapy with both of these approaches showed a decline in the count of major bleeding episodes on follow-up. Overall, albeit both methods have proven useful, LAAO has a slight advantage in efficacy and leads to less hemorrhagic events.

## Introduction and background

It is calculated that almost 7% of individuals older than 65 years and 15-20% in the age bracket of 80-89 years are affected by atrial fibrillation (Afib) [1]. Fibrillating atrium may cause stasis of blood and activation of coagulation, which may elevate the risk of thromboembolism; this leads to an overall risk of stroke of 5% every year [1]. The most common position of the left atrial appendage (LAA) is between the anterior and lateral walls of the left atrium, with its tip directed anterosuperiorly, extending over the left border of the pulmonary trunk [2]. The LAA has many lobes and pouches; their number in the LAA is directly proportional to the risk of thrombus formation in patients with Afib [3]. The left atrium is curved and has smooth walls; hence, thrombi do not form there. The LAA, on the other hand, has a diverse structure; it is blind-ended and has a trabeculated meshwork formed by the pectinate muscles [1]. Afib can broadly be divided into two categories, valvular and non-valvular, in etiology [4]. The favored therapy for valvular Afib is vitamin K antagonism, but trials have demonstrated an essential role for percutaneous LAA occlusion (LAAO) in non-valvular Afib [4].

Oral anticoagulation (OAC) has proven to be quite useful in individuals with non-valvular Afib. Until recently, the only means for the treatment of Afib related stroke was vitamin K antagonists (VKA). Now non-vitamin K oral anticoagulants (NOACs) are also available, such as dabigatran, rivaroxaban, apixaban, and edoxaban [5]. Despite their effectiveness, patients with perceived or absolute contraindication to OACs due to the risk of bleeding cannot benefit from this therapy. For these patients, LAAO has emerged as a nonpharmacological alternative for the prevention of stroke [6,7]. The two devices most commonly used in clinical practice are the Watchman® and Amulet® (Amplatzer) devices, but randomized studies are only available for the Watchman device [8].

Currently, long-term studies are being conducted to evaluate the complications of post-procedural effects on patients undergoing LAAO by a multitude of devices. Many new devices have emerged lately. Future studies should be directed toward how to stratify patients into subgroups based on individual characteristics so that they can be directed towards the LAAO device, which would be the most effective and lead to the least harmful side effects in the long run.

The purpose of this review is to compare the two most common methods used in the prevention of stroke due to non-valvular Afib (OAC and LAAO) and to evaluate which is more effective.

## Review

Discussion

At present, more than 7% of people aged 65 and above are affected by Afib [1]. It is essential that the best treatment options are clear and defined so that the patients and physicians can select treatment options with the assurance of better results. This review article will be a comparison of two commonly used methods of stroke prevention in patients with non-valvular Afib and will try to conclude which of the two is more clinically safe in the short- and long-term prevention of stroke. The main objective is to help physicians make a better choice between these treatment options and to improve patient morbidity and mortality.

Safety and Efficacy of OAC and LAAO in the Prevention of Stroke in Patients with Non-Valvular Afib

**Figure 1:**
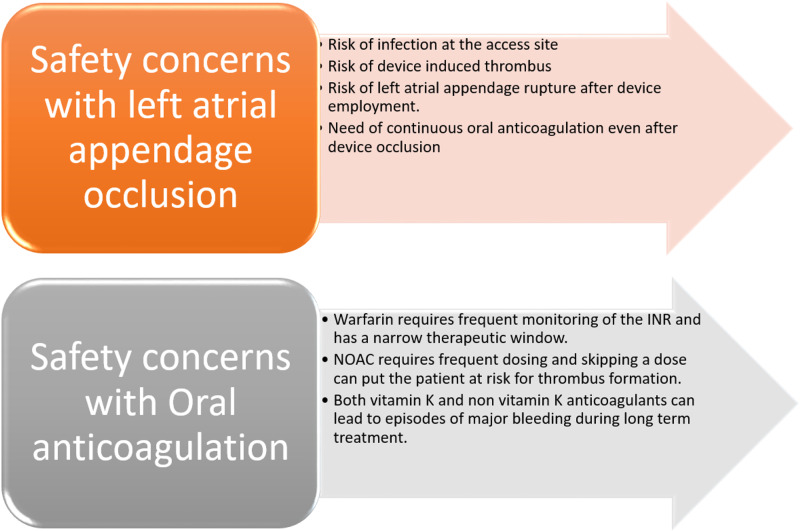
Safety Concerns with LAAO and OAC LAAO, left atrial appendage occlusion; OAC, oral anticoagulation

For ages, OAC has been the mainstay of treatment for stroke prevention in patients with thromboembolic events and was accepted worldwide as the standard of treatment. VKAs were used most commonly but required constant monitoring; this proved to be a hindrance as it needed more management hours, a short therapeutic window, and higher overall cost. An alternative in the form of NOACs was introduced, which was superior in that it required less monitoring and had higher but not clinically significant safety and efficacy levels. All types of OAC treatments, however, confer a risk of bleeding in the long term. This was an important issue that has been addressed by the most advanced treatment approach of LAAO through endocardial and epicardial devices, which have shown to reduce the rate of bleeding in the long run. All of the particular treatment modalities have their safety concerns (Figure 1).

Interestingly, a network meta-analysis conducted by Hanif et al. concluded that LAAO, when compared to warfarin, aspirin, or placebo, was the most efficacious treatment when it came to the outcomes of stroke [9]. A separate study conducted by Sahay et al. also proposed the same result but with a more substantial degree of evidence as it focused on a larger group of patients. It suggested that as there was no statistically significant difference in the outcomes of warfarin and LAAO. Still, when compared to NOAC, a lower mortality rate was seen as antiplatelet therapy, and placebo had worse mortality outcomes [10]. Thus, we can infer that the overall most efficacious treatment is LAAO, but it only has a slight edge over warfarin. The treatment option of NOAC should be considered because of lower mortality rates, albeit it resulted in high rates of gastrointestinal bleeding compared to LAAO. Neither of the studies focused on age-related outcomes, which could lead to better decisions and clinical outcomes. Future studies need to stratify patients into age groups and focus on the consequences of these interventions.

Although LAAO seems to be the better alternative even by a small margin, some studies oppose the notion of LAAO being superior to OAC. A retrospective case-control study conducted in 2018 with 124 patients in 22 years showed that the exclusion of LAA did not reduce early or late stroke. It went a step further to propose that patients on OAC had better outcomes [11]. However, a study was conducted by Sharma et al. that studied different LAAO devices, and their findings showed that LAAO devices were adequate for the prevention of stroke [12]. This study is a review; hence, it has precedence over the previously mentioned case-control study based on the premise that the study has a higher quality of evidence. It also was limited in that the sample size was relatively small. Hence, it would be reasonable to propose that LAAO devices lead to a similar if not better outcome when it comes to stroke prevention compared to OAC. Numerous other studies also support this argument [8,13,14]. To date, the most aggressively evaluated LAAO device is the Watchman device. Randomized controlled trials are needed to assess other LAAO devices further.

In most cases, OAC therapy has shown to be effective. However, there are still rare individuals in which, despite anticoagulation, episodes of stroke or transient ischemic attack occur, and LAAO might be of benefit for such cases. A study that came out in 2020 put forward the proposition that in patients with resistant stroke, LAAO leads to a reduction in episodes of stroke during follow-up [15]. Another study by Pouru et al. in the same year also showed the same results [16]. Both studies used Amplatzer devices. However, the study by Pouru et al. had a small number of patients included and was a single-center study; therefore, there is a considerable chance of selection bias. The observation of the effect these devices in high-risk individuals who have had episodes of stroke and cannot take OAC is a relatively new venture for researchers, and there is a significant gap in the understanding of the outcomes. Future researchers need to focus on observational studies with larger groups of patients to come to a conclusion (Table 1).

**Table 1:** Safety and Efficacy of OAC and LAAO in the Prevention of Stroke LAAO, left atrial appendage occlusion; OAC, oral anticoagulation; NOAC, non-vitamin K oral anticoagulation

Author	Year of publication	Purpose of study	Intervention studied	Conclusion
Hanif et al. [9]	2018	To analyze different randomized control trials; comparing LAAO to the standard of care in patients with atrial fibrillation	LAAO and OAC	Although there was no significant difference, LAAO showed to be more safe and efficacious when it was compared to warfarin, aspirin, or placebo as a treatment for stroke
Sahay et al. [10]	2017	To assess the safety and efficacy of LAAO compared to other strategies of stroke prevention	LAAO and OAC	It was shown that LAAO had superiority over placebo and antiplatelet therapy and showed no significant difference when compared to NOAC
Johnsrud et al. [11]	2018	It was done to evaluate the need of OAC after LAAO	LAAO and OAC	It undermined LAAO and proposed that LAAO had no significant effect in the reduction of stroke, whereas OAC was associated with a reduction in stoke
Sharma et al. [12]	2018	To show the safety and efficacy of different LAAO devices	LAAO	The Watchman device, Amplatzer plug, Lariat occlusion system, and Atri clip show favorable data and support their usage in a patient with atrial fibrillation
Masoud et al. [13]	2018	To see if LAAO could be used as an alternative to standard treatment in patients who were at high risk	LAAO	In patients in whom OAC in contraindicated, LAAO may be a reasonable option
Tereshchenko et al. [14]	2016	To compare the safety and efficacy of different interventions in non-valvular atrial fibrillation	OACs (vitamin K and non-vitamin K) and Watchman device	It was found that all anti-embolic intervention significantly reduced stroke, but the two most effective interventions were LAAO and NOAC
Hutt et al. [8]	2020	Assess the role of the Watchman device in patients who are at high risk for stroke	Watchman device	In patients who had a high risk of stroke, the implementation of the Watchman device proved to be both safe and efficacious
Cruz-González et al. [15]	2020	To analyze the safety of LAAO in patients who have had a previous episode of stroke	Amplatzer cardiac plug	It was seen that patients with stroke despite OAC after undergoing LAAO showed no difference compared to patients who did not undergo this intervention
Pouru et al. [16]	2020	To evaluate LAAO in patients with previous thromboembolism	LAAO	In patients with previous episodes of intracranial bleeding, LAAO demonstrated to be a viable option

Long-Term Efficacy of Therapies on Stroke Rate

After the two modalities of treatment had been introduced, LAAO and OAC, there remained the question of which of these two would be the best choice in long-term therapy and lead to lesser complications, improving patient safety and lifespan in the long haul. To answer this question, two-course changing trials, PREVAIL (Evaluation of the Watchman LAA Closure Device in Patients With Atrial Fibrillation Versus Long Term Warfarin Therapy) and PROTECT AF (Watchman Left Atrial Appendage System for Embolic Protection in Patients With Atrial Fibrillation) trials, were carried out, both over five-year-long spans. In 2017, a group of individuals collectively analyzed both of these trials and their results, concluding that the Watchman device is non-inferior to warfarin in the prevention of stroke caused by non-valvular Afib. However, one added benefit of using the Watchman device was that it reduced the episodes of major bleeding and also lead to an improvement in the mortality rate [17]. In recent years, a study by Litwinowicz et al. focused on the long-term outcome of an epicardial approach to LAAO with the Lariat device in high-risk individuals with a contraindication to OAC and showed that the Lariat device was a viable and safe alternative to OAC [18]. A paper published by Regueiro et al. in 2018 supported the idea of LAAO in another prospective study arguing the benefit of long-term use of LAAO devices. They mainly focused on the Amplatzer cardiac plug, showing its effectiveness and safety over a term of five years [19]. Analyzing the above studies, it can be inferred that although the devices proved to be non-inferior to OAC, the studies conducted for the evaluation of these devices included only small groups of people. The device studied in the most number of people was the Watchman device, but that too needs further evaluation. The studies on Lariat and the Amplatzer cardiac plug were single-center studies, although they support the notion of the use of LAAO devices in high-risk patients. To date, the most reliable LAAO device in the long term is the Watchman device; a paper put forward by Wiebe et al. is also in favor of the long-term use of the Watchman device [20]. There is still a void that needs filling by studies that have a higher quality of evidence. Future studies should focus on compiling these small individual long-term trials in the form of a meta-analysis to improve the quality of evidence (Table 2).

**Table 2:** Long-Term Safety and Efficacy of LAAO and OAC PREVAIL, Evaluation of the Watchman LAA Closure Device in Patients With Atrial Fibrillation Versus Long Term Warfarin Therapy; PROTECT AF, Watchman Left Atrial Appendage System for Embolic Protection in Patients With Atrial Fibrillation; LAAO, left atrial appendage occlusion; OAC, oral anticoagulation

Author	Year of publication	Purpose of study	Intervention studied	Conclusion
Reddy et al. [17]	2017	To review the results of the PREVAIL trial and the PROTECT AF trial	Watchman device	It showed that LAAO with the Watchman device is comparable to warfarin in patients who have atrial fibrillation with the additional benefit of reducing major bleeding
Litwinowicz et al. [18]	2018	To assess the long-term outcome in patients with occlusion using the Lariat device	Lariat device	The Lariat device showed to be a safe and effective treatment for stroke prevention in high-risk patients having atrial fibrillation
Regueiro et al. [19]	2018	To analyze the long-term outcomes in patients who had a contraindication to OAC and used LAAO as an alternative	Amulet device and the Watchman device	LAAO is safe in the long haul in patients who had a contraindication to anticoagulation
Wiebe et al. [20]	2015	Evaluation of the long-term outcome in patients with atrial fibrillation who had undergone percutaneous LAAO with the Watchman device	Watchman device	LAAO with the Watchman device showed low rates of ischemic events in the long-term follow-up

Combined Therapy and its Outcome

To increase the quality of patient care and to hopefully find a new avenue of treatment, combined therapy with LAAO and OAC was tried, which gave positive results. A prospective study using this combination of therapy on patients with the previous stroke came out in support of combined therapy, arguing the effectiveness of this treatment method. Patients with indefinite OAC and LAAO were observed, and no episodes of major bleeding were seen in follow-up [21]. Another study by Freixa et al. supports the same notion that it is more reliable than the prior mentioned study because the trial group was larger [22]. But both came to the same endpoint, strengthening the argument in favor of this combined method.

A multicenter prospective study was conducted to evaluate the theory of combined therapy focusing mainly on the usage of the Watchman device as the LAAO modality showed that when combined with anticoagulation therapy, no significant episodes of major bleeding were seen. Of all the OACs used, the ones with the lowest bleeding rate were the NOACs [23]. In 2015, Seeger et al. put forward an argument that supported this idea and further vouches for the viability of a dual combination method [24]. Albeit this study is less reliable compared to the aforementioned study because it is not as recent, still they are both in agreement. It is quite apparent that there is a benefit of the combination of OAC with LAAO. However, there is still a need to aggressively study dual therapy in randomized controlled trials to decrease bias and also to conduct long-term studies assessing their benefits (Table 3).

**Table 3:** The Outcome of Combination Therapy OAC, oral anticoagulation; LAAO, left atrial appendage occlusion; LAA, left atrial appendage; DAPT, dual antiplatelet therapy; VKA, vitamin K antagonists; NOAC, non-vitamin K oral anticoagulants

Author	Year of publication	Purpose of the study	Intervention studied	Conclusion
Masjuan et al. [21]	2019	To evaluate the safety and efficacy of combining OAC and LAAO in patients with recurrent strokes despite satisfactory anticoagulation	LAAO and OAC	The outcome of indefinite OAC with LAAO in patients who had recurrent stroke showed to be beneficial in patients with previous ischemic attacks
Freixa et al. [22]	2019	Studying LAAO in patients with stroke despite optimal OAC	LAAO and OAC	LAAO used as an adjunctive therapy to OAC showed to decrease the rate of cerebrovascular accidents
Bergmann et al. [23]	2017	To observe the outcome of various drugs after the implantation of the Watchman device	Watchman device	After the closure of the LAA, when patients received DAPT, VKA, and NOAC, it was seen that the additional therapy had no added benefit
Seeger et al. [24]	2016	To observe the effect of LAAO in patients with non-valvular atrial fibrillation having high bleeding risk	Watchman device or the Amplatzer plug along with OAC	LAAO in patients with non-valvular atrial fibrillation and a high risk of bleeding prevented stroke occurrence

Limitations

This review is subject to limitations due to the lack of studies with a higher degree of evidence; this has led to the inevitable inclusion of selection bias as most of the studies were observational studies. This review is deficient in that not many randomized controlled trials have been conducted with larger cohorts. Hence, it is challenging for physicians to choose the best modality of treatment as there is no clearcut evidence.

Furthermore, there is a lack of studies in patients with varying degrees of risk factors, which is very important while considering LAAO or OAC; therefore, a clear answer to the question of which treatment option would benefit which group of patients could not be given.

We had a few other limitations such as not many devices have been compared to OAC for the prevention of stroke. The Watchman device has been studied and has a convincing degree of evidence for its usage, but other useful methods have not been considered as often. Clinical trials with the intervention of different LAAO devices would have strengthened this study. These are needed to solidify the base of making a decision.

## Conclusions

This review focused on a comparison between two of the most widely used treatments for stroke prevention in patients with non-valvular Afib, OAC and LAAO. LAAO was shown to have greater efficacy than OACs, although when compared with VKA, the difference was not significant. For long-term treatment, LAAO is non-inferior to OAC. Combined therapy in high-risk patients leads to fewer episodes of major bleeding. This review has made it easier for future clinicians to make a more informed decision as to which modality of treatment will be beneficial to their patients in the short and long term. It also opens to a window to try combination therapy in cases of recurrent stroke. Future studies need to focus on large-scale randomized trials to reduce bias, guiding the way to better treatment plans for these high-risk individuals.
